# Synergistic Antioxidant Activity and Enhanced Stability of Curcumin Encapsulated in Vegetal Oil-Based Microemulsion and Gel Microemulsions

**DOI:** 10.3390/antiox11050854

**Published:** 2022-04-27

**Authors:** Cristina Scomoroscenco, Mircea Teodorescu, Sabina Georgiana Burlacu, Ioana Cătălina Gîfu, Catalin Ionut Mihaescu, Cristian Petcu, Adina Raducan, Petruta Oancea, Ludmila Otilia Cinteza

**Affiliations:** 1 Polymer Department, National Institute for Research and Development in Chemistry and Petrochemistry-ICECHIM, 202 Spl. Independentei, 060021 Bucharest, Romania; scomoroscencocristina@gmail.com (C.S.); sabina.nitu@yahoo.com (S.G.B.); gifu_ioanacatalina@yahoo.com (I.C.G.); mihaescu_catalin96@yahoo.com (C.I.M.); cpetcu@icf.ro (C.P.); 2 Faculty of Applied Chemistry and Materials Science, University Politehnica of Bucharest, 010737 Bucharest, Romania; mircea.teodorescu@upb.ro; 3 Physical Chemistry Department, University of Bucharest, 030018 Bucharest, Romania; adina.raducan@g.unibuc.ro (A.R.); petruta.oancea@unibuc.ro (P.O.)

**Keywords:** curcumin, microemulsion, grape seed oil, synergism, antioxidant activity

## Abstract

Curcumin, due to its antioxidant, antibacterial, anti-inflammatory, and antitumoral activity, has attracted huge attention in applications in many fields such as pharmacy, medicine, nutrition, cosmetics, and biotechnology. The stability of curcumin-based products and preservation of antioxidant properties are still challenges in practical applications. Stability and antioxidant properties were studied for curcumin encapsulated in O/W microemulsion systems and three related gel microemulsions. Only biodegradable and biocompatible ingredients were used for carriers: grape seed oil as oily phase, Tween 80, and Plurol^®^ Diisostearique CG as a surfactant mix, and ethanol as a co-solvent. For the gel microemulsions, water-soluble polymers, namely Carbopol^®^ 980 NF, chitosan, and sodium hyaluronate were used. The influence of UVC irradiation and heat treatment on the degradation kinetics of curcumin in the formulations was studied. Because of the antioxidant character of the microemulsion oily phase, the possibility of a synergistic effect between grape seed oil and curcumin was explored. In this study, the high efficiency of the studied drug delivery systems to ensure protection from external degradative factors was confirmed. Also, the influence of the encapsulation in microemulsion and derived gel microemulsion systems on the antioxidant capacity curcumin was studied, and a synergistic effect with vegetal oil was demonstrated.

## 1. Introduction

Curcumin (CURC) is a polyphenolic compound that has been recognized for its medicinal properties for decades, as it is used both in cuisine as a spice in its natural form or as an extract in pharmaceutical or cosmetic formulations [1]. Curcumin and its derivatives, demethoxycurcumin, and bisdemethoxycurcumin, exhibit several beneficial properties and proved to be effective in wound healing, treatment of inflammatory diseases, obesity, diabetes, novel synergistic anticancer therapies joint with current chemotherapeutics, innovative preventive medicine in neurodegenerative diseases [2]. In addition to its potential uses in pharmaceutical products, curcumin is considered a valuable nutraceutical [3]. In addition, because of the bright yellow color, curcumin is used as a natural pigment in the food, cosmetic, and pharmaceutical industries [4]. However, research continues to further develop the potential of curcuminoids in various fields, especially where their antioxidant activity is exploited [5].

The highly hydrophobic nature of curcumin determines its low water solubility, and thus, limited bioavailability and cellular uptake, which is the major bottleneck in clinical applications. Another significant drawback is the susceptibility to external degradative factors, such as thermal and ultraviolet radiation [6]. To increase aqueous solubility, stability, and pharmacokinetic properties, different formulations for the encapsulation of CURC have been proposed and investigated, such as liposomes, polymeric or lipid nanoparticles hydrogels, biopolymer complexes, or emulsions [7,8,9]. Microemulsions have been recognized as potent drug delivery systems due to their large amount of oily content able to encapsulate curcuminoids compared to other colloidal carriers. In particular, microemulsions, due to their unique characteristics of thermodynamic stability, very small liquid droplet size, and convenient fabrication were extensively studied as vehicles for the enhancement of oral or transdermal delivery of curcumin [10,11]. Additionally, the encapsulation of curcuminoids in microemulsions has been reported as an efficient method to increase the stability of the sensitive natural compounds, as it is proved by UV-Vis and fluorescence investigations [12]. Liu et al. [10] prepared a surfactant-free microemulsion containing ethyl benzoate, water, and ethanol with O/W structure, that allowed high curcumin encapsulation, up to 17.54 mg/mL and resulted in an increase of antioxidant activity. In another study, Rahdar et al. [11] reported the encapsulation of curcumin in an O/W microemulsion based on ethyl butyrate as an oily phase and stabilized with Pluronic F127 and sodium caprilate. The CURC-loaded microemulsion exhibited an EE larger than 88%, prolonged release of the drug, and high antitumoral activity.

Gel microemulsions—as nanostructured systems obtained by adding thickening agents in aqueous or oily phases of a microemulsion—combine the benefits from the physico-chemical characteristics of both gels and microemulsions. The main advantages of the pharmaceutical and cosmetic formulations based on gel microemulsion have enhanced stability and easy topical administration, compared to simple microemulsions, due to increased viscosity [13].

The most important reason for the large spread of the use of CURC in pharmaceuticals, supplements, and functional foods is its significant antioxidant activity. Its efficiency as an antioxidant in various media, from food and cosmetic product to living organisms, is due to a multi-component mechanism that involves the ability to act as a free radical scavenger and singlet oxygen quencher. It is also proven that it could chelate ferric (Fe^3+^) and ferrous (Fe^2+^), known as pro-oxidants, in human cells [14].

Numerous studies reported enhanced antioxidant properties of the curcumin encapsulated in colloidal vectors such as, liposomes [15], emulsions [16,17], polymeric microparticles [18,19], hydrogel beads [20], nanoparticles [21], and microemulsions [10,11].

The further improvement of the beneficial biomedical properties of curcumin has been pursued through several approaches. One way is the synthesis of novel derivatives with various pharmacologically active groups grafted onto the curcuminoid molecules to obtain compounds with increased antitumor, antibacterial, or antioxidant activity [22]. Another possibility is to combine other natural or synthetic active substances with curcumin to potentiate a desired biological effect, as in combined chemotherapy with CURC and synthetic anticancer drugs co-encapsulated in nanoparticulated DDS, which show promising results in killing multidrug-resistant tumoral cells [23]. Additionally, many papers report enhanced antimicrobial efficiency when curcumin is administrated together with antibiotics or nanoparticles with antibacterial properties [24]. The combination of CURC with other compounds to achieve a higher level of antioxidant activity was less investigated.

Most of the documented cases of synergism in curcuminoides—other therapeutics mixtures are related to the antitumoral or antibacterial efficiency [25], while a limited number of studies have emphasized the synergism in antioxidant behavior. Moreover, very few papers reporting synergism have actually provided quantitative analysis due to difficulties in using the mathematical methods to compute the synergetic effects in mixtures with natural compounds [26].

As in the case of other drugs, the majority of the published papers refer to the synergistic effects recorded in the combination of curcumin with another active ingredient co-encapsulated in the same carrier [27,28] but only occasionally treat the synergistic effects with the components of the delivery system [29].

In this work, the stability and antioxidant properties of curcumin encapsulated in one microemulsion and three derived gel microemulsions obtained in a previous study [30] are presented. The oil-in-water microemulsion system and related gel microemulsions reported have been obtained using only natural components, as formulation proposed for topical administration of curcumin for cosmetic purposes. The development of microemulsion formulations, together with the encapsulation efficiency and kinetics of the drug release, were reported in our previous paper [30].

Due to the presence of cold-pressed grape seed oil as the oily phase of the microemulsion, which also exhibits antioxidant properties [31,32], we assumed that synergism may occur between curcumin and the vegetable oil. Considering the unique composition of the developed microemulsion and gel microemulsion systems, in which curcumin is encapsulated, in this paper, for the first time, the effect of the vegetable oily phase on the stability and antioxidant properties is presented and the synergistic effect of curcumin–grape seed oil was proved.

## 2. Materials and Methods

### 2.1. Materials

Curcumin (95%, Natures aid), DPPH (2,2-diphenyl-1-picrylhydrazyl, Sigma-Aldrich, St. Louis, MO, USA), quercetin hydrate (≥95%, Sigma-Aldrich), cold-pressed grape (*Vitis vinifera*) seed oil (MAYAM Cosmetics, Oradea, Romania), ethanol absolute (>99.5%, Chimreactiv, Bucharest, Romania,), Plurol^®^ Diisostearique CG (polyglyceryl-3 diisostearate, kindly gifted by Gattefosse, Saint-Priest, France), Tween 80 (Sigma-Aldrich), Carbopol^®^ 980 NF (high molecular weight poly(acrylic acid), (Lubrizol Advanced Materials Inc., Cleaveland, OH, USA), sodium hyaluronate salt (*Streptococcus equi* sp., molecular weight ~1.5–1.8 × 10^6^ Da, Sigma-Aldrich), chitosan (highly viscous, Fluka, Buchs, Switzerland) were used as received. All solvents used for HPLC analysis, acetonitrile (>99.9%), phosphoric acid solution (>85%), water, and ethanol absolute (>99.8%) were purchased from Sigma-Aldrich and were HPLC grade purity. Distilled water was used in all experiments.

### 2.2. Preparation of Microemulsion and Gel Microemulsion for Curcumin Encapsulation

One optimized microemulsion and three derived gel microemulsions were prepared as drug delivery systems (DDS) for the encapsulation of CURC. The composition of these carriers and the conditions of preparing the samples were optimized in our previously published paper [30] in order to obtain a suitable formulation for cosmetic application. The selected microemulsion contained grape seed oil as oily phase (7.6%), water (23.7%), surfactant–cosurfactant mixture Tween 80 and Plurol^®^ Diisostearique CG (45.2 and 15.1%, respectively), and ethanol as the co-solvent (8.4%), expressed as % from the entire mass of the nanosystem.

For the preparation of microemulsion, oil grape seed, water, ethanol, and surfactants Tween 80 and Plurol^®^ Diisostearique CG in the above-mentioned ratios were added in a vial and mixed by using Vortex equipment for 1 minute. The system was left to equilibrate for 48 h at 25 °C. To achieve a higher viscosity, gel microemulsions were prepared by adding to the previously described microemulsion three polymers Carbopol^®^ 980 NF, sodium hyaluronate, and chitosan, as thickening agents. In order to obtain gel microemulsions with Carbopol^®^ 980 NF and hyaluronic acid sodium salt, the solid polymers were added to the microemulsion. The system was left at least 48 h under vigorous magnetic stirring, until complete dissolution of the polymer. To obtain the gel microemulsion modified with chitosan, a polymeric solution in 1% acetic acid was prepared and further used as an aqueous phase for the microemulsion preparation. Thus, the other microemulsion components (grape seed oil, surfactant mixture, co-solvent ethanol) were added to the chitosan solutions previously prepared, and the system was homogenized by using mini Vortex equipment. The polymer concentrations in the gel microemulsions, expressed as mass percentage based on the entire microemulsion mass, was fixed as 0.2 wt.% for Carbopol^®^ 980 NF, 0.34 wt.% for chitosan, and with 0.3 wt.% for sodium hyaluronate, based on the optimization reported in the previous study [30].

Curcumin encapsulation was carried out by adding the solid powder to the microemulsion and derived gel microemulsions prepared as described above. The CURC- loaded systems were subjected to vigorous magnetic stirring for 24–48 h until the complete dissolution of the drug.

### 2.3. Characterization of Microemulsions and Gel Microemulsions

The liquid droplet size in microemulsion was measured by using the DLS method, on a Zetasizer Nano ZS instrument (Malvern Instruments, Malvern, UK) equipped with a 532 nm He-Ne laser and a scattering angle of 173°. Conductivity measurements were performed using undiluted samples using a Cole-Parmer 500 conductivity meter (Cole Parmer, Vernon Hills, IL, USA). The rheological properties of the microemulsion-based gels were investigated using a Kinexus Pro rheometer (Malvern Instruments, Malvern, UK) with Peltier element for temperature control. The measurements were carried out at 25 °C, in rotation mode, using a 1°/40 mm cone.

### 2.4. Stability Study

The stability of the curcumin encapsulated in grape seed oil-based microemulsion (ME) and three gel microemulsions was investigated.

The degradation of curcumin in microemulsion (ME), gel microemulsions (g-ME), and control systems was achieved by irradiation with UVC light, at 254 nm at room temperature or by heat treatment at 37 and 80 °C, using the adapted methodology from the literature [4,33,34]. Table 1 shows the name of the samples subjected to the degradation study and the amount of curcumin in each sample.

For photodegradation experiments, a low-pressure Hg lamp, UVC type, with an emitted wavelength of 254 nm, with a power of 50 W (Philips), was used. A volume of 1.5 mL of each sample, undiluted and covered with a lid to prevent evaporation of the sample, was irradiated in a quartz cuvette. The cuvette was placed at a distance of 2 cm from the UVC lamp.

For thermal degradation, a volume of 1.5 mL of each sample, undiluted, was placed in well-sealed glass vials on a water bath, which maintained the samples at 37 °C and 80 °C, respectively.

As a control, the remaining amount of each sample was kept in the dark in the refrigerator. For both methods, the samples were degraded for 2 h. High-performance reverse-phase liquid chromatography (HPLC) was used to determine the amount of curcumin in the samples before and after degradation. For all samples, except the US sample, for which no dilution was required, a 1:100 mass dilution with ethanol was applied. HPLC analyses were run on a Jasco LC-NetII/ADC HPLC with a Jasco UV–2075Plus Intelligent UV–VIS detector, set at 420 nm, and a Jasco PU-2089Plus Quaternary Gradient Pump. A Teknokroma HPLC NUCLEOSIL 100 C-18 5 μm (25 cm × 0.4 cm) column was selected. The mobile phase consisted of phosphoric acid solution (0.1 vol.%) and acetonitrile in a volumetric ratio of 1 to 1. The flow rate was 1 mL/min and the column temperature was set at 25 °C. In these conditions, the retention time of curcumin is 9.1 min.

The percentage of degraded curcumin was calculated using the following equation:

(1) $$\mathrm{CRD}\%=100-\frac{C_{\mathrm{final}}}{C_{\mathrm{initial}}}\times 100$$

where C_final_—the amount of curcumin after degradation, and C_initial_—the amount of curcumin before degradation.

### 2.5. In Vitro Antioxidant Activity of the Encapsulated Curcumin

The stability of the in vitro antioxidant activity of curcumin in the systems of microemulsion and gel microemulsions was studied by the method based on measuring the wavelength absorbance of the specific maximum for 2,2-diphenyl-1-picrylhydrazyl (DPPH). The antioxidant, in this study curcumin, has the role of reducing DPPH free radicals that in ethanol forms a dark purple color, and thus, the DPPH solution turns yellow. Quantification of the percentage of reduced DPPH by curcumin was performed by reading the absorbance at 517 nm using a UV-VIS spectrophotometer (Jasco V-530, Tokyo, Japan).

The samples analyzed to determine the antioxidant activity of curcumin were M1, ME, SM, gME-CT, gME-SH, and gME-CP, whose composition and quantity of curcumin are indicated in Table 1. Curcumin dissolved in ethanol was used as a reference for CURC antioxidant activity in standard solution, and quercetin solution was employed as a positive control. The method described in the literature [4,11,35] was adapted according to the specific characteristics of the studied samples. A stock solution of DPPH with a concentration of 40 mg/L was used. A volume of 2.5 mL of solution was analyzed on a UV-VIS spectrophotometer, of which 10 μL was sampled, and the rest was a stock solution of DPPH. For the stock solution of DPPH, absorbance was recorded and the resulting value represented the absorbance at time t = 0 (A_0_), for each of the samples. For sample M1, the absorbance was recorded, beginning 10 s after mixing the DPPH stock solution with the sample. For the rest of the samples, the absorbance was recorded beginning with 20 s; because of high viscosity, a longer time was needed to ensure the homogeneity of the final solution. Thus, this aspect was taken into account in the graphic representations and time corrections were made. The absorbance was recorded by the spectrophotometer every 2 s for 900 s.

The antioxidant capacity of curcumin was expressed as the percentage of reduced DPPH free radicals. This was calculated using the following equation [11]:

(2) $$\%\;{\mathrm{DPPH}}_{\mathrm{reduced}}=\frac{A_{0}-\mathrm{As}}{A_{0}}\times 100$$

where A_0_—the initial absorbance of DPPH solution without CURC (negative control), and A_s_—the absorbance of DPPH solution with the antioxidant after 900 s.

### 2.6. Synergistic Effects in Antioxidant Activity of Curcumin–Grape Seed Oil Mixtures

#### 2.6.1. DPPH Free Radical-Scavenging Capacity Assay for Different Curcumin-Grape Seed Oil Ratios

Antioxidant efficiency of curcumin and grape seed oil apart towards DPPH was evaluated by monitoring the absorbance decrease at 517 nm for 900 s. The percentage of the reduced DPPH was calculated using Formula (2) and was noted as % ESC-experimental scavenging capacity [3,36].

The quantity of curcumin and grape seed oil was the same as the quantity used in each corresponding curcumin–grape seed oil mixtures.

#### 2.6.2. Calculation of Synergistic Effect (SE) of Curcumin–Grape Seed Oil Mixtures

DPPH assay was used to evaluate the interaction between curcumin and grape seed oil in the mixtures,. Five mixtures of grape seed oil and curcumin were prepared by adding curcumin ethanolic solution 0.5 wt. % to grape seed oil; the proportion of grape seed oil:curcumin ranging between 1:10 to 10:1 (in volume). For the 1:1 proportion, grape seed oil concentration was 18 mg/mL and for curcumin concentration was 79 μg/mL.

The theoretical scavenging capacity (% TSC) is the sum of the scavenging capacities of each antioxidant, calculated using the individual scavenging capacity in the following equation [37]:

(3) $$\%\;\mathrm{TSC}={\%\;\mathrm{ESC}}_{\mathrm{oil}}+{\%\;\mathrm{ESC}}_{\mathrm{curc}}-\frac{{\%\;\mathrm{ESC}}_{\mathrm{oil}}\times {\%\;\mathrm{ESC}}_{\mathrm{curc}}}{100}$$

where % ESC_oil_ and % ESC_curc_—experimental scavenging capacity of the individual antioxidant.

The synergetic effect (SE) of the combination of curcumin and grape seed oil was calculated based on the ratios of the experimental and theoretical scavenging capacities using the following equation:

(4) $$\mathrm{SE}=\frac{\%\;\mathrm{ESC}}{\%\;\mathrm{TSC}}$$

The synergetic effect is present if SE is greater than 1 [38].

### 2.7. Statistical Analysis

All experiments were performed in triplicate, and the results were presented as mean and standard deviation ± SD. Differences between the antioxidant activity of CURC in various formulations were assessed by one-way ANOVA, using MS Excel 2010 from Microsoft (Redmond, WA, USA). Statistical significance was considered at *p* < 0.05.

## 3. Results and Discussions

### 3.1. Preparation and Characterization of Micoremulsion and Gel Microemulsions Loaded with Curcumin

Microemulsion and gel microemulsions with curcumin encapsulated were prepared as nanostructured carriers formulated with selected ingredients for cosmetic application, as described in our previous paper [30]. CURC was encapsulated to a high extent (1–2%) in the microemulsion, and related gel microemulsion systems at the highest concentration, as previously determined [30].

**Figure 1:**
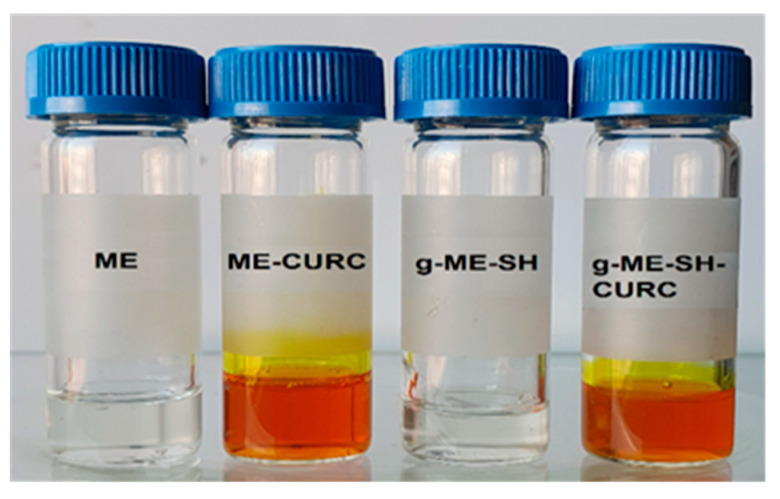
Visual aspect of microemulsion and gel microemulsion with sodium hyaluronate (see Table 1) with and without encapsulated CURC.

The sample, with and without the drug, were homogeneous and transparent, as one could observe in the optical images of some selected microemulsion-based drug delivery systems (simple microemulsions and gel microemulsions with sodium hyaluronate as the thickening agent) in Figure 1.

For the parent microemulsion, a value of electrical conductivity of 220 μS/cm was recorded that suggests that the obtained system is an O/W microemulsion [39].

**Figure 2:**
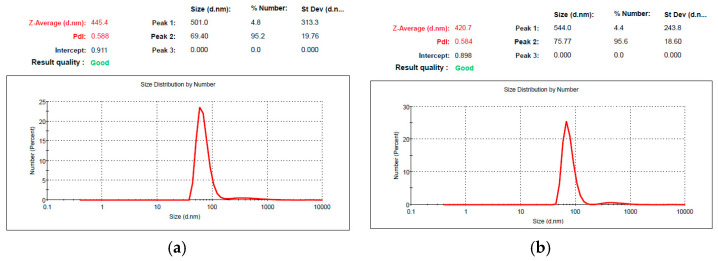
Size and size distribution for void microemulsion (**a**) and curcumin encapsulating microemulsion (**b**).

The size and size distribution of the oily droplets in the freshly prepared microemulsions with and without CURC are presented in Figure 2.

The size of the droplets in the microemulsion was 71.44 ± 1.69 nm (mean value calculated from measurements of 3 samples), and the encapsulation of CURC did not significantly change the dimension since the average value in the microemulsion with 1% drug was 72.79 ± 9.21 nm.

The detailed microemulsion and gel microemulsion rheological characterization was discussed in our previous published paper [30], where the results demonstrated that ME showed a Newtonian behavior and the gel microemulsions showed a pseudoplastic non-Newtonian rheological behavior, similar to other reported gel microemulsions.

Based on the conclusions of our previous rheological study, gel microemulsion systems containing a suitable amount of polymer in the aqueous phase, that allow the maximum enhancement of the viscosity were selected for this work. The viscosity at a shear rate of 10 (s^−1^) was 0.36 Pa∙s for the parent microemulsion, while the values determined for gel microemulsions were 0.61 Pa∙s for the g-ME with 0.2 wt.% Carbopol^®^ 980 NF, 0.72 Pa∙s for the g-ME with 0.34 wt.% chitosan, and 1.05 Pa∙s for the g-ME with 0.3 wt.% sodium hyaluronate.

### 3.2. Stability of Curcumin Encapsulated in Gel Microemulsions

One of the limitations in the application of curcumin is its sensitivity to heat and radiation, resulting in chemical degradation and, consequently, in the reduction of biological properties. Thus, the goal of the encapsulation of CURC in a suitable carrier is to ensure the proper vehicle to administrate for biomedical use, but also to allow better protection of the active ingredient.

The stability of encapsulated curcumin was further investigated for the optimized microemulsion and the three gel microemulsions in relation to some reference samples, i.e., curcumin incorporated in ethanol, the mix of surfactants, and grape seed oil, in order to evidence the specific effect of different components.

The percentage of curcumin degraded after 2 h of exposure to different degradation factors (irradiation with UVC light and heat treatment at 37 and 80 °C) are shown in Table 2. In the samples kept as controls, in the dark and in the refrigerator, the amount of curcumin remained unmodified during the same period of time (data not shown). Thus, the percentage of degraded curcumin was strictly due to irradiation, respectively the heat treatment applied to the samples for 2 h.

As it is expected, the high temperature (80 °C) produces the most important degradation of curcumin compared to the effect of exposure to UVC light and lower temperature (37 °C). These results are consistent with the conclusions reported in the literature [40,41].

The highest amount of degraded curcumin was recorded for samples M1 and SM, i.e., curcumin incorporated in ethanol, respectively, in the surfactant mix. Over a quarter of the curcumin encapsulated in ethanol was degraded after a 2 h exposure of the sample to 80 °C. Curcumin dissolved in the grape seed oil (sample OP) suffered insignificant degradation. Although curcumin has been protected from grape seed oil, a disadvantage is the much lower solubility of curcumin in the oil compared to the ability of microemulsion to encapsulate curcumin. In microemulsion and gel microemulsions, a very small amount of curcumin was degraded by heat treatment at 80 °C. According to the literature, curcumin subjected to heat treatment can generate modifications, such as a shift of the double bonds, degradation to lower molecular weight compounds as vanillin, vanillic acid, and ferulic acid. The transformations mentioned above denote indicating that the weak point of heated curcumin is the “diketone bridge” [42].

In the microemulsion-based samples exposed to UVC light and heat treatment at 37 °C, the amount of curcumin remained in most cases unmodified, demonstrating the high efficiency of the microemulsion and gel microemulsions systems to protect curcumin from external degradative factors. The exception is the sample in microemulsion (ME), which exhibits a 4% degradation of CURC after exposure to UVC while still showing no degradation under thermal exposure at 37 °C. Thus, the developed gel microemulsion delivery systems were found to ensure the enhanced stability of the curcumin and protection against either thermal or UVC radiation exposure.

### 3.3. Antioxidant Activity of CURC Encapsulated in Microemulsion and Gel Microemulsion Systems

Most of its uses in pharmaceuticals and cosmetics are based on the antioxidant properties of CURC, so it is crucial that the formulation used for encapsulation be able to ensure that the antioxidant activity is maintained.

The antioxidant capacity of curcumin encapsulated in microemulsion and derived gel microemulsions was compared with the antioxidant capacity in the control samples, i.e., curcumin solubilized in ethanol (M1) and the surfactant mix (SM).

**Figure 3:**
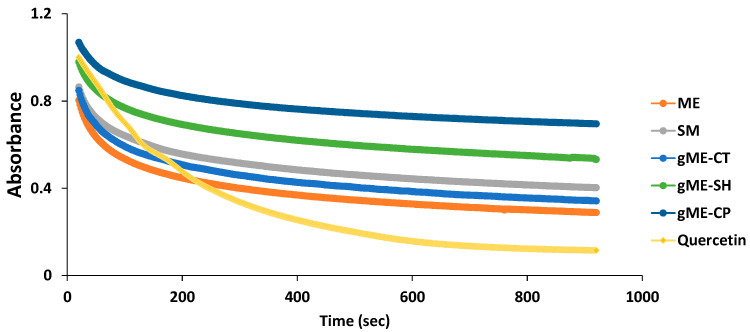
Evolution of the DPPH solution absorbance for CURC encapsulated in various microemulsion systems and reference solvent mixtures (sample denoted in Table 1).

The time variation of the free radical reduction capacity of curcumin encapsulated in various drug delivery systems expressed as a decrease in absorbance of the DPPH sample, is shown in Figure 3.

From Figure 3, it can be seen that the highest amount of DPPH is reduced in the first 100 s, then up to t_f_ of 900 s a plateau is formed. For this reason, the absorbance at t_f_ = 900 s was considered to be directly correlated with the total amount of reduced DPPH free radicals.

**Figure 4:**
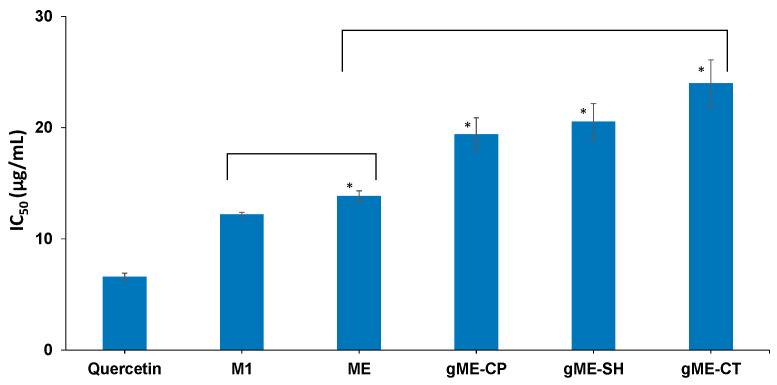
IC_50_ values of CURC encapsulated in microemulsion, gel microemulsions systems, and reference solvent mixtures (sample denoted in Table 1). (*) statisticaly significant (*p* < 0.05).

The samples concentration providing 50% inhibition of free radicals (IC_50_) was calculated and are represented in Figure 4. A lower value for IC_50_ is consistent with greater antioxidant activity.

Quercetin was used in the study as a positive control since it is known as a flavonoid compound with high antioxidant activity. The determined IC_50_ for the quercetin sample in the experimental conditions was found as 6.60 ± 0.31 μg/mL, a value similar to those reported in the literature [43]. IC_50_ for curcumin in ethanol solution is 12.22 ± 0.16 μg/mL, and the value is comparable with data reported in other research papers [44].

The encapsulation of CURC in the optimized microemulsion (sample ME) leads to a slightly but statistically significant increase in IC_50_ value (13.85 ± 0.48 μg/mL) compared to CURC in ethanolic solution. Further decrease in the antioxidant activity is also noticed after encapsulation in gel microemulsions, with IC_50_ values 19.41 ± 1.47 μg/mL for gME-CP, 20.54 ± 1.63 μg/mL for gME-SH, and 24.00 ± 2.10 μg/mL for gME-CT, respectively. No statistically significant differences were found in the antioxidant activities of CURC embedded in the gel microemulsions formulated with various polymers, thus, the chemical composition of the gelators does not affect the capacity to reduce the free radicals. The small decrease of the antioxidant capacity of curcumin in the gel microemulsions is probably due to the good entrapment of curcumin inside the droplets of the nanostructured systems. In other studies, a slight decrease of the antioxidant activity was also reported as result of encapsulation in complex nanosystems [45] as compared to CURC in ethanolic solution.

**Figure 5:**
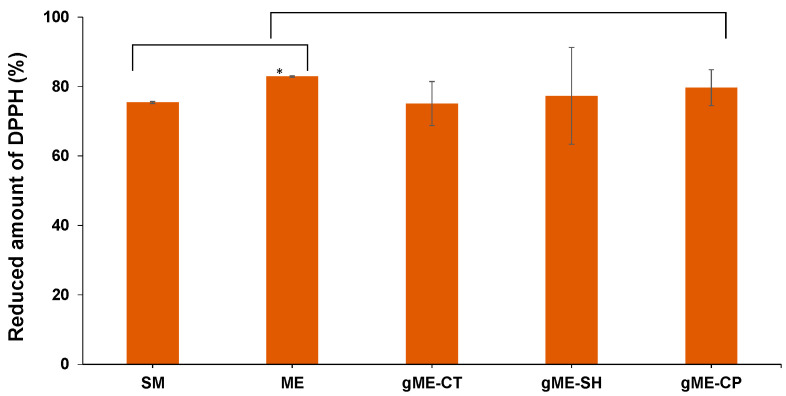
Reduced amount of DPPH, for CURC encapsulated in various microemulsion systems (at 1% concentration) and reference solvent mixtures (sample denoted in Table 1) determined at equilibrium (time = 900 s). (*) statisticaly significant (*p* < 0.05).

The obtained CURC-loaded gel microemulsions were developed as formulations for cosmetic applications [30]; thus, the preservation of the antioxidant activity in the samples with the highest encapsulation of active compound was targeted. The reduction of DPPH free radicals was compared for samples with a specific concentration of more than 10 mg/mL CURC, a concentration close to the maximal drug solubilization determined in the drug delivery systems (Figure 5).

As was expected, the percentage of reduced DPPH by the microemulsion without curcumin was very small, 3.2% (data not represented in Figure 5). According to the microemulsion composition, it is presumable that the measured antioxidant activity is due to the presence of the grape seed oil.

The highest amount of DPPH reduction, about 82%, was recorded for CURC encapsulated in the optimized microemulsion (ME sample), a statistically significant increased value compared to the sample of CURC dissolved in the mixture of surfactants (SM). Still, the value is smaller than the one recorded for the positive control quercetin (89.57%) due to the intrinsic antioxidant activity of curcumin, which is known to be inferior to the quercetin.

Similar values were obtained for the free radical reduction of curcumin encapsulated in gel microemulsions, i.e., 77.30% in gel microemulsion sodium hyaluronate (g-ME_SH), 75.09% in gel microemulsion with chitosan, and 79.65% in gel microemulsion with Carbopol^®^ 980 NF. No significant difference was found between the antioxidant activities of CURC encapsulated in gel microemulsions, regardless of the chemical nature of the polymer, compared to the active compound encapsulated in the original microemulsion.

Thus, adding water-soluble polymer to the optimized microemulsion in order to obtain gel microemulsions with suitable viscosity for topical application of cosmetic product, does not produce a considerable change in the antioxidant capacity of the active compound curcumin.

Although the antioxidant mechanism of curcumin is not fully explained, keto-enol tautomerism and solvation are considered the most important factors in terms of the antioxidant activity of curcumin [46]. Experimental [47,48] and theoretical [49] studies indicate that curcumin is predominantly in enol form in non-polar environments, respectively, predominantly in keto form in polar solvents. So, the antioxidant capacity might be based on the specific π-conjugated structure with two o-methoxyphenols, existing in the enol form of curcumin [46]. These facts may be a viable explanation for the variation of the antioxidant activity of curcumin in ethanol and in the microemulsion system, where CURC could be located in the oily phase, in the water-alcoholic phase, and in the surfactant film at the oil-water interface.

These results have demonstrated the preservation of most of the antioxidant activity of the curcumin encapsulated in the optimized microemulsion and gel microemulsions compared to the behavior in ethanolic solution.

### 3.4. Synergistic Effect between Curcumin and Grape Seed Oil

In addition to curcumin, grape seed oil from the composition of the microemulsion is the only ingredient that has antioxidant activity. Since an increase in antioxidant activity for microemulsion and gel microemulsion samples was recorded compared to the solution of CURC in the mixture of surfactant, it was necessary to conduct a study to evaluate the antioxidant activity of the combination of vegetable oil used as oily phase and curcumin.

#### 3.4.1. Antioxidant Activity of Single Compounds

**Figure 6:**
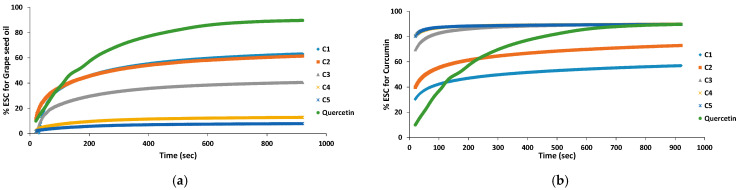
Variation of the % ESC for individual components Grape seed oil (**a**) and for curcumin (**b**) at various concentrations equivalent to concentrations at the selected grape seed oil-curcumin mixtures investigated for synergistic effect, at different ratio (*v*/*v*): C1 corresponds to 10:1 (*v*/*v*) ratio; C2 corresponds to 4.5:10 (*v*/*v*) ratio; C3 corresponds to 1:1 (*v*/*v*) ratio; C4 corresponds to 1:5 (*v*/*v*) ratio; C5 corresponds to 1:10 (*v*/*v*) ratio.

The changes in the scavenging capacity of curcumin and grape seed oil measured with the DPPH free radical are shown in Figure 6. Curcumin is a very efficient antioxidant, the scavenging capacity being over 50% at a concentration of 14 μg/mL. By comparison, for a similar antioxidant activity for grape seed oil, its concentration must be around 30 mg/mL. For both curcumin and grape seed oil, after 15 min the absorbance of DPPH remains almost constant; the reaction appears to be dose-dependent for both antioxidants (Figure 6), the scavenging activity reaching a maximum at a certain antioxidant concentration ([ao]).

For the estimation of the sample concentration providing 50% inhibition (IC_50_), a nonlinear regression curve was fitted to experimental data [50,51]:

(5) $$\%\;\mathrm{ESC}=a+b\left(1-e^{-c\left[\mathrm{ao}\right]}\right)$$

**Figure 7:**
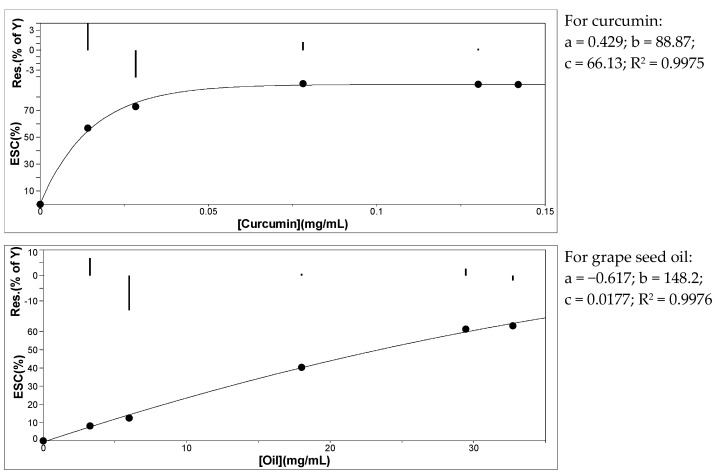
Variation of experimental scavenging activity toward DPPH with antioxidant concentration (solid lines represent the best fit of Equation (5) to experimental data).

The results were obtained by using ORIGIN 8.0 software [51], and the values for IC_50_ are 23.5 ± 1.4 mg/mL for oil and 12.3 ± 0.2 μg/mL for curcumin (Figure 7).

It is obvious that curcumin is a much better antioxidant than grape seed oil, IC_50_ for curcumin was found to be almost 2000 lower than the IC_50_ for grape seed oil.

#### 3.4.2. Synergistic Effect (SE) of Curcumin–Grape Seed Oil Mixtures

To investigate the existence of synergistic effect in grape seed oil–curcumin mixtures, the free radical scavenging capacities of several antioxidant combinations were studied, denoted as experimental scavenging capacity of oxidant mixture. Based on the data obtained from the individual antioxidants, the theoretical scavenging capacities (% TSCs) were calculated at times 30 s and 900 s. If the experimental values are the same as the theoretical ones, then the contribution of the individual antioxidant would be additive. If the % ESC of the mixture is greater than the TSC value, then an interaction happens among the antioxidants, thus displaying synergism. The results are presented in Table 3.

The results indicate that the concentration of the individual antioxidants has a direct effect on SE. At the beginning of the reaction (t_1_ = 30 s), for all tested mixtures, the grape seed oil produced a synergetic effect, even in very low quantities. At longer times (t_2_ = 900 s), after the reaction reaches the equilibrium, the synergetic effect is observed only for the mixtures with high content of oil (for the oil-curcumin solution ratios beyond 4.5:1).

The synergetic effect in the grape seed oil–curcumin mixtures appears at a relatively high amount of oil and does not lead to an extensive increase (only 5.5 %) in the antioxidant activity, compared to theoretical value. The reduction of DPPH free radicals produced by grape seed oil–curcumin mixtures is still smaller than the value recorded for the positive control quercetin, as is expected since both curcumin and grape seed oil are significantly less effective as antioxidants.

However, the use of natural oils, such as grape seed oil, in the preparation of microemulsions as carriers for active ingredients proved to be beneficial in improving the antioxidant activity of cosmetic formulations.

## 4. Conclusions

The aim of this study was to evaluate the stability and antioxidant properties of curcumin encapsulated in a microemulsion and three derived gel microemulsions formulated with natural ingredients as oily phase and gelation polymers. In addition, because of the presence in microemulsion systems of the cold-pressed grape seed oil, which also has antioxidant properties, the possible synergistic effect of curcumin–grape seed oil mixtures was examined.

The degradation of CURC encapsulated in microemulsion and gel microemulsions exposed to the most common stressors (irradiation with UVC light and temperatures of 37 and 80 °C), it was possible to show the high efficiency of the systems to protect curcumin from external degradative factors.

The increased stability of curcumin encapsulated in optimized microemulsion and gel microemulsions was confirmed, with results comparable to the stability reported in other microemulsions [52].

The encapsulation of CURC in microemulsion and derived gel microemulsion systems results in a slight decrease in the antioxidant activity (IC_50_ values decrease compared to CURC in ethanolic solution) without significant changes related to the type of the polymer used as a thickening agent. However, at a high degree of drug encapsulation in the microemulsion-based drug delivery systems, CURC still retains high DPPH reduction capacity, up to 82% in microemulsion and 79.65% in gel microemulsion with Carbopol^®^ 980 NF.

The scavenging capacity of curcumin (as an ethanolic solution) and grape seed oil measured individually indicate that curcumin is more efficient antioxidant, IC_50_ for curcumin being almost 2000 lower than the IC_50_ for grape seed oil. For the first time, the occurrence of synergism in the curcumin–grape seed oil mixture was evidenced. In the early stage of the free radical inhibition (t_1_ = 30 s) the grape seed oil, even in very low quantities, produced a synergetic effect for all tested curcumin–oil mixtures, while after the reaction’s equilibrium is reached, the synergetic effect is observed only when the oil–curcumin solution ratios exceed 4.5:1 (in volume).

This study shows the high efficiency of optimized microemulsion and gel microemulsions systems to protect curcumin from external degradative factors and preserve the antioxidant stability of CURC. Those results indicate the perspective of using the proposed gel-microemulsions loaded with curcumin for further development of cosmetic and nutraceutical products, together with the possibility of using them for the possible protective action on other sensitive pharmacological active compounds.

## Figures and Tables

**Table 1:** Sample ID and amount of curcumin encapsulated in each of the samples.

Sample ID	Encapsulating System/Solvent	Amount of Curcumin in Sample (mg/mL)
M1	Ethanol	3.95
ME	Microemulsion	10.13
SM	Polyglyceryl-3 Diisostearate:Tween 80 weight ratio 1:3 (surfactant mixture)	10.6
OP	Grape seed oil (oily phase of ME)	0.28
gME-CP	Gel microemulsion with Carbopol^®^ 980 NF	10.13
gME-SH	Gel microemulsion with sodium hyaluronate	10.13
gME-CT	Gel microemulsion with chitosan	10.13

**Table 2:** The amount of curcumin degraded after 2 h, after exposure at UVC, 37 °C, and 80 °C.

Sample ID	Degraded Curcumin (%)		
	UVC (254 nm)	80 °C	37 °C
M1	19.47	26.32	17.95
SM	16.80	16.12	4.12
OP	0.12	0.43	0
ME	4.03	4.71	0
gME-CP	0	3.69	0
gME-SH	1.54	3.52	0
gME-CT	0	8.50	0

**Table 3:** Synergistic effect (SE) and experimental scavenging capacity (% ESC) of antioxidant mixtures at 30 s and 900 s.

Oil:Curcumin Solution (0.5 wt.% in Ethanol)	t_1_ = 30 s			t_2_ = 900 s		
	% ESC_1_	% TSC_1_	SE_1_	% ESC_2_	% TSC_2_	SE_2_
1:10	85.37 ± 0.04	83.37 ± 0.05	* 1.024 ± 0.001	89.41 ± 0.04	90.37 ± 0.06	0.989 ± 0.001
1:5	82.45 ± 0.07	80.77 ± 0.18	* 1.020 ± 0.002	89.86 ± 0.02	90.55 ± 0.04	0.992 ± 0.005
1:1	76.10 ± 0.01	71.70 ± 0.07	* 1.061 ± 0.002	91.39 ± 0.07	93.81 ± 0.11	0.974 ± 0.016
4.5:1	60.92 ± 0.36	58.09 ± 0.37	* 1.048 ± 0.027	90.79 ± 0.48	90.13 ± 0.51	1.007 ± 0.023
10:1	45.76 ± 0.28	43.99 ± 0.21	* 1.041 ± 0.009	89.29 ± 0.39	84.68 ± 0.56	* 1.054 ± 0.025

Theoretical scavenging capacity (TSC) was calculated from data for single components (see Figure 6); * Asterisks denote a significant difference compared with the respective experimental value (*p* < 0.05).

## Data Availability

Data is contained within the article.

## References

[1] Kocaadam B., Şanlier N. (2017). Curcumin, an Active Component of Turmeric (*Curcuma longa*), and Its Effects on Health. Crit. Rev. Food Sci. Nutr..

[2] Salehi B., Stojanović-Radić Z., Matejić J., Sharifi-Rad M., Anil Kumar N.V., Martins N., Sharifi-Rad J. (2019). The Therapeutic Potential of Curcumin: A Review of Clinical Trials. Eur. J. Med. Chem..

[3] Kunnumakkara A.B., Bordoloi D., Padmavathi G., Monisha J., Roy N.K., Prasad S., Aggarwal B.B. (2017). Curcumin, the Golden Nutraceutical: Multitargeting for Multiple Chronic Diseases. Br. J. Pharmacol..

[4] Artiga-Artigas M., Lanjari-Pérez Y., Martín-Belloso O. (2018). Curcumin-Loaded Nanoemulsions Stability as Affected by the Nature and Concentration of Surfactant. Food Chem..

[5] Jakubczyk K., Drużga A., Katarzyna J., Skonieczna-Żydecka K. (2020). Antioxidant Potential of Curcumin—A Meta-Analysis of Randomized Clinical Trials. Antioxidants.

[6] Kharat M., Du Z., Zhang G., McClements D.J. (2017). Physical and Chemical Stability of Curcumin in Aqueous Solutions and Emulsions: Impact of PH, Temperature, and Molecular Environment. J. Agric. Food Chem..

[7] Gong F., Chen D., Teng X., Ge J., Ning X., Shen Y., Li J., Wang S. (2017). Curcumin-Loaded Blood-Stable Polymeric Micelles for Enhancing Therapeutic Effect on Erythroleukemia. Mol. Pharm..

[8] Grilc N.K., Sova M., Kristl J. (2021). Drug Delivery Strategies for Curcumin and Other Natural Nrf2 Modulators of Oxidative Stress-Related Diseases. Pharmaceutics.

[9] Stohs S.J., Chen O., Ray S.D., Ji J., Bucci L.R., Preuss H.G. (2020). Highly Bioavailable Forms of Curcumin and Promising Avenues for Curcumin-Based Research and Application: A Review. Molecules.

[10] Liu W., Pan N., Han Y., Li D., Chai J. (2021). Solubilization, Stability and Antioxidant Activity of Curcumin in a Novel Surfactant-Free Microemulsion System. LWT.

[11] Rahdar A., Hajinezhad M.R., Sargazi S., Zaboli M., Barani M., Baino F., Bilal M., Sanchooli E. (2021). Biochemical, Ameliorative and Cytotoxic Effects of Newly Synthesized Curcumin Microemulsions: Evidence from In Vitro and In Vivo Studies. Nanomaterials.

[12] Mondal S., Ghosh S., Moulik S.P. (2016). Stability of Curcumin in Different Solvent and Solution Media: UV–Visible and Steady-State Fluorescence Spectral Study. J. Photochem. Photobiol. B.

[13] Telò I., Favero E.D., Cantù L., Frattini N., Pescina S., Padula C., Santi P., Sonvico F., Nicoli S. (2017). Gel-like TPGS-Based Microemulsions for Imiquimod Dermal Delivery: Role of Mesostructure on the Uptake and Distribution into the Skin. Mol. Pharm..

[14] Sun Y.-M., Zhang H.-Y., Chen D.-Z., Liu C.-B. (2002). Theoretical Elucidation on the Antioxidant Mechanism of Curcumin: A DFT Study. Org. Lett..

[15] Ali N., Choudhury S.T., Das N., Ghosh S., Ghosh D., Chakraborty S. (2016). Vesicular (Liposomal and Nanoparticulated) Delivery of Curcumin: A Comparative Study on Carbon Tetrachloride–Mediated Oxidative Hepatocellular Damage in Rat Model. Int. J. Nanomed..

[16] Pan Y., Tikekar R.V., Nitin N. (2013). Effect of Antioxidant Properties of Lecithin Emulsifier on Oxidative Stability of Encapsulated Bioactive Compounds. Int. J. Pharm..

[17] Carpenter J., George S., Saharan V.K. (2019). Curcumin Encapsulation in Multilayer Oil-in-Water Emulsion: Synthesis Using Ultrasonication and Studies on Stability and Antioxidant and Release Activities. Langmuir.

[18] Gómez-Estaca J., Balaguer M.P., López-Carballo G., Gavara R., Hernández-Muñoz P. (2017). Improving Antioxidant and Antimicrobial Properties of Curcumin by Means of Encapsulation in Gelatin through Electrohydrodynamic Atomization. Food Hydrocoll..

[19] Fırtın B., Yenipazar H., Saygün A., Şahin-Yeşilçubuk N. (2020). Encapsulation of Chia Seed Oil with Curcumin and Investigation of Release Behaivour & Antioxidant Properties of Microcapsules during in Vitro Digestion Studies. LWT.

[20] Wang H., Gong X., Guo X., Liu C., Fan Y.-Y., Zhang J., Niu B., Li W. (2019). Characterization, Release, and Antioxidant Activity of Curcumin-Loaded Sodium Alginate/ZnO Hydrogel Beads. Int. J. Biol. Macromol..

[21] Pontes-Quero G.M., Benito-Garzón L., Pérez Cano J., Aguilar M.R., Vázquez-Lasa B. (2021). Amphiphilic Polymeric Nanoparticles Encapsulating Curcumin: Antioxidant, Anti-Inflammatory and Biocompatibility Studies. Mater. Sci. Eng. C.

[22] Khor P.Y., Aluwi M.F.F.M., Rullah K., Lam K.W. (2019). Insights on the Synthesis of Asymmetric Curcumin Derivatives and Their Biological Activities. Eur. J. Med. Chem..

[23] Batra H., Pawar S., Bahl D. (2019). Curcumin in Combination with Anti-Cancer Drugs: A Nanomedicine Review. Pharmacol. Res..

[24] Perera W.P.T.D., Dissanayake R.K., Ranatunga U.I., Hettiarachchi N.M., Perera K.D.C., Unagolla J.M., De Silva R.T., Pahalagedara L.R. (2020). Curcumin Loaded Zinc Oxide Nanoparticles for Activity-Enhanced Antibacterial and Anticancer Applications. RSC Adv..

[25] Hosseini-Zare M.S., Sarhadi M., Zarei M., Thilagavathi R., Selvam C. (2021). Synergistic Effects of Curcumin and Its Analogs with Other Bioactive Compounds: A Comprehensive Review. Eur. J. Med. Chem..

[26] Cinteza L.O., Scomoroscenco C., Voicu S.N., Nistor C.L., Nitu S.G., Trica B., Jecu M.-L., Petcu C. (2018). Chitosan-Stabilized Ag Nanoparticles with Superior Biocompatibility and Their Synergistic Antibacterial Effect in Mixtures with Essential Oils. Nanomaterials.

[27] De Paz-Campos M.A., Ortiz M.I., Piña A.E.C., Zazueta-Beltrán L., Castañeda-Hernández G. (2014). Synergistic Effect of the Interaction between Curcumin and Diclofenac on the Formalin Test in Rats. Phytomedicine.

[28] Cui Y., Zhang M., Zeng F., Jin H., Xu Q., Huang Y. (2016). Dual-Targeting Magnetic PLGA Nanoparticles for Codelivery of Paclitaxel and Curcumin for Brain Tumor Therapy. ACS Appl. Mater. Interfaces.

[29] Manca M.L., Castangia I., Zaru M., Nácher A., Valenti D., Fernàndez-Busquets X., Fadda A.M., Manconi M. (2015). Development of Curcumin Loaded Sodium Hyaluronate Immobilized Vesicles (Hyalurosomes) and Their Potential on Skin Inflammation and Wound Restoring. Biomaterials.

[30] Scomoroscenco C., Teodorescu M., Raducan A., Stan M., Voicu S.N., Trica B., Ninciuleanu C.M., Nistor C.L., Mihaescu C.I., Petcu C. (2021). Novel Gel Microemulsion as Topical Drug Delivery System for Curcumin in Dermatocosmetics. Pharmaceutics.

[31] Shinagawa F.B., de Santana F.C., Torres L.R.O., Mancini-Filho J. (2015). Grape Seed Oil: A Potential Functional Food?. Food Sci. Technol..

[32] Fernandes L., Casal S., Cruz R., Pereira J.A., Ramalhosa E. (2013). Seed Oils of Ten Traditional Portuguese Grape Varieties with Interesting Chemical and Antioxidant Properties. Food Res. Int..

[33] Chen C., Johnston T.D., Jeon H., Gedaly R., McHugh P.P., Burke T.G., Ranjan D. (2009). An in Vitro Study of Liposomal Curcumin: Stability, Toxicity and Biological Activity in Human Lymphocytes and Epstein-Barr Virus-Transformed Human B-Cells. Int. J. Pharm..

[34] Ansari M.J., Ahmad S., Kohli K., Ali J., Khar R.K. (2005). Stability-Indicating HPTLC Determination of Curcumin in Bulk Drug and Pharmaceutical Formulations. J. Pharm. Biomed. Anal..

[35] Greenwald M.B.Y., Frušić-Zlotkin M., Soroka Y., Sasson S.B., Bitton R., Bianco-Peled H., Kohen R. (2017). Curcumin Protects Skin against UVB-Induced Cytotoxicity via the Keap1-Nrf2 Pathway: The Use of a Microemulsion Delivery System. Oxid. Med. Cell. Longev..

[36] Quiroga P.R., Nepote V., Baumgartner M.T. (2019). Contribution of Organic Acids to α-Terpinene Antioxidant Activity. Food Chem..

[37] Liu D., Shi J., Ibarra A.C., Kakuda Y., Xue S.J. (2008). The Scavenging Capacity and Synergistic Effects of Lycopene, Vitamin E, Vitamin C, and β-Carotene Mixtures on the DPPH Free Radical. LWT Food Sci. Technol..

[38] Wong F.-C., Xiao J., Ong M.G.-L., Pang M.-J., Wong S.-J., Teh L.-K., Chai T.-T. (2019). Identification and Characterization of Antioxidant Peptides from Hydrolysate of Blue-Spotted Stingray and Their Stability against Thermal, PH and Simulated Gastrointestinal Digestion Treatments. Food Chem..

[39] Kale S.N., Deore S.L. (2016). Emulsion Micro Emulsion and Nano Emulsion: A Review. Syst. Rev. Pharm..

[40] Li Z., Peng S., Chen X., Zhu Y., Zou L., Liu W., Liu C. (2018). Pluronics Modified Liposomes for Curcumin Encapsulation: Sustained Release, Stability and Bioaccessibility. Food Res. Int..

[41] Syed H.K., Liew K.B., Loh G.O.K., Peh K.K. (2015). Stability Indicating HPLC–UV Method for Detection of Curcumin in Curcuma Longa Extract and Emulsion Formulation. Food Chem..

[42] Paramera E.I., Konteles S.J., Karathanos V.T. (2011). Stability and Release Properties of Curcumin Encapsulated in Saccharomyces Cerevisiae, β-Cyclodextrin and Modified Starch. Food Chem..

[43] Li X., Jiang Q., Wang T., Liu J., Chen D. (2016). Comparison of the Antioxidant Effects of Quercitrin and Isoquercitrin: Understanding the Role of the 6″-OH Group. Molecules.

[44] Wei L., Li X., Guo F., Liu X., Wang Z. (2018). Structural Properties, in Vitro Release and Radical Scavenging Activity of Lecithin Based Curcumin-Encapsulated Inverse Hexagonal (HII) Liquid Crystals. Colloids Surf. Physicochem. Eng. Asp..

[45] Kaur K., Kaur J., Kumar R., Mehta S.K. (2017). Formulation and Physiochemical Study of α-Tocopherol Based Oil in Water Nanoemulsion Stabilized with Non Toxic, Biodegradable Surfactant: Sodium Stearoyl Lactate. Ultrason. Sonochem..

[46] Ohara K., Mizukami W., Tokunaga A., Nagaoka S., Uno H., Mukai K. (2005). Kinetic Study of the Mechanism of Free-Radical Scavenging Action in Curcumin: Effects of Solvent and PH. Bull. Chem. Soc. Jpn..

[47] Tonnesen H.H., Karlsen J., Henegouwen G.B. (1986). Studies on Curcumin and Curcuminoids VIII. Photochemical Stability of Curcumin. Z. Lebensm. Unters. Forsch..

[48] Bubbly S.G., Gudennavar S.B., Verghese B., Viswam D., Sudarsanakumar C. (2008). Crystal Structure of 1,7-Bis(4-Chlorophenyl)-4-(1,3-Dithiolan-2-Ylidene)-1,6-Heptadiene-3,5-Dione. J. Chem. Crystallogr..

[49] Wright J.S. (2002). Predicting the Antioxidant Activity of Curcumin and Curcuminoids. J. Mol. Struct. Theochem.

[50] Re R., Pellegrini N., Proteggente A., Pannala A., Yang M., Rice-Evans C. (1999). Antioxidant Activity Applying an Improved ABTS Radical Cation Decolorization Assay. Free Radic. Biol. Med..

[51] Polak J., Bartoszek M. (2018). A New Equation for Converting the Parameter EC50 into the Total Antioxidant Capacity TEAC and Vice Versa. Food Chem..

[52] Sintov A.C. (2015). Transdermal Delivery of Curcumin via Microemulsion. Int. J. Pharm..

